# Impact of light and nutrient availability on the phagotrophic activity of harmful bloom-forming dinoflagellates

**DOI:** 10.1093/plankt/fbae038

**Published:** 2024-07-17

**Authors:** Catalina Mena, Marc Long, Ophélie Lorand, Pascale Malestroit, Emilie Rabiller, Jean-François Maguer, Stéphane L’helguen, Aurore Regaudie De Gioux

**Affiliations:** Écologie Pélagique (DYNECO/PELAGOS), Institut Français de Recherche pour l’Exploitation de la Mer, IFREMER, 29280 Plouzané, France; Centre Oceanogràfic de Balears, Instituto Español de Oceanografía, COB-IEO, CSIC, Moll de Ponent s/n, 07015 Palma (Illes Balears), Spain; Écologie Pélagique (DYNECO/PELAGOS), Institut Français de Recherche pour l’Exploitation de la Mer, IFREMER, 29280 Plouzané, France; University of Brest, CNRS, IRD, IFREMER, Laboratoire des sciences de l'environnement marin (LEMAR), F-29280 Plouzané, France; Écologie Pélagique (DYNECO/PELAGOS), Institut Français de Recherche pour l’Exploitation de la Mer, IFREMER, 29280 Plouzané, France; Écologie Pélagique (DYNECO/PELAGOS), Institut Français de Recherche pour l’Exploitation de la Mer, IFREMER, 29280 Plouzané, France; Écologie Pélagique (DYNECO/PELAGOS), Institut Français de Recherche pour l’Exploitation de la Mer, IFREMER, 29280 Plouzané, France; University of Brest, CNRS, IRD, IFREMER, Laboratoire des sciences de l'environnement marin (LEMAR), F-29280 Plouzané, France; University of Brest, CNRS, IRD, IFREMER, Laboratoire des sciences de l'environnement marin (LEMAR), F-29280 Plouzané, France; Écologie Pélagique (DYNECO/PELAGOS), Institut Français de Recherche pour l’Exploitation de la Mer, IFREMER, 29280 Plouzané, France

**Keywords:** phagotrophy, mixotrophy, bloom-forming dinoflagellates, nutrient limitation, light limitation

## Abstract

Phagotrophy is a key nutritional mode for many bloom-forming dinoflagellates that can supplement their carbon and nutrient requirements. However, the environmental drivers and ecological relevance of phagotrophy in algal blooms are still poorly understood. This study evaluates the effect of light and nutrient availability on the phagotrophic activity of three common bloom-forming dinoflagellates (*Alexandrium minutum*, *Heterocapsa triquetra* and *Prorocentrum micans*) using three fluorescently labeled preys: bacteria, *Synechococcus* and the haptophyte *Isochrysis galbana*. The three dinoflagellates exhibited distinct responses to light and nutrient availability in terms of growth, cell size, prey ingestion and preference. *A. minutum* and *H. triquetra* showed higher cell-specific ingestion rates on bacteria (0.53 ± 0.13 and 1.64 ± 0.39 prey dinoflagellate^−1^ h^−1^, respectively) under co-limited nutrient and light availability, whereas *P. micans* showed higher ingestion on *Synechococcus* (0.93 ± 0.22 prey dinoflagellate^−1^ h^−1^) under low-light availability alone. However, the three dinoflagellates exhibited the highest carbon and nitrogen-specific ingestion rates when feeding on the larger prey *I. galbana*. Our findings indicate that phagotrophy could be of advantage in short periods of light or nutrient limitation and may play different roles during the development of blooms, likely influencing the energy transfer through the food web.

## INTRODUCTION

Mixotrophy is a ubiquitous nutritional strategy among marine protist plankton that combines autotrophy and heterotrophy in the same cell (Burkholder *et al.,* 2008; Mitra *et al.,* 2014; Selosse *et al.,* 2017; Stoecker *et al.,* 2017). Mixotrophic protists have the innate capability for inorganic carbon (C) fixation through photosynthesis and also the ability to acquire C from organic sources (Mitra *et al.,* 2016). Different strategies of heterotrophic uptake are used, such as osmotrophy (i.e. absorption through cell membranes of dissolved organic compounds), tube-feeding (i.e. using a peduncle to suck part or the entire plasmatic content of preys) or phagotrophy (i.e. engulfment and digestion of organic particles or preys into the cell by phagocytosis), among others (reviewed by Selosse *et al.,* 2017). It is the last type, i.e. photo-phago-trophy, that we will consider here, also referred to as mixoplankton or constitutive mixotrophs in the literature (Flynn *et al.,* 2019; Glibert and Mitra, 2022; Mitra *et al.,* 2023).

Model simulations suggest that mixotrophy can significantly enhance the C transfer to higher trophic levels in the food web, increase nutrient retention in surface waters and the C flux to the deep ocean (Mitra *et al.,* 2014; Ward and Follows, 2016; Stoecker *et al.,* 2017), with important consequences for the ocean C cycle and atmospheric C sequestration. However, the identification of mixotrophic species has been expanded in the last few decades and the full scope of their ecological importance remains poorly understood (Mitra *et al.,* 2023). The increasing number of studies on mixotrophy in the last years has revealed that many phototrophic dinoflagellates have also phagotrophic capacity (Jacobson and Andersen, 1994; Stoecker, 1999; Jeong *et al.,* 2010; Mitra *et al.,* 2023) and that most of harmful algal blooms of coastal and eutrophic waters are caused by phago-mixotrophic dinoflagellates (Jeong *et al.,* 2005b; Burkholder *et al.,* 2008; Jeong *et al.,* 2021). This highlights the potential importance phagotrophy may have as nutrition mode in bloom dynamics and the need to better understand its environmental or biotic drivers.

Phagotrophy is suggested to supplement C and nutrient requirements when photosynthesis is limited due to low light or deficient nutrient concentrations (Stoecker, 1999; Unrein *et al.,* 2007). However, studies to date show different results on the effect of nutrient or light availability on phagotrophic activity, suggesting that the environmental conditions that favor phagotrophy may vary among species (Jeong *et al.,* 2010; Berge *et al.,* 2012; Yang *et al.,* 2020). Some studies observed that nutrient depletion can trigger or enhance phagotrophy of blooming algae, e.g. *Phaeocystis globosa* (Koppelle *et al.,* 2022), *Heterocapsa triquetra* (Legrand *et al.,* 1998) and *Tripos furca* (previously identified as *Ceratium furca*) (Smalley *et al.,* 2003), which indicate that under nutrient-limiting conditions, such as during late stage of blooms, mixotrophs can obtain C and nutrients from prey ingestion. Nonetheless, Zhang *et al.* (2013) reported no apparent relationship between nutrient conditions and the phagotrophic activity of other blooming dinoflagellates such as *Karenia mikimotoi*, *Alexandrium catenella*, *Prorocentrum donghaiense* and *Prorocentrum micans*. The effect of light level is also variable in the response of mixotrophic dinoflagellates. Indeed, prey ingestion can increase under low or limiting light conditions in some species (Hansen, 2011; Smalley *et al.,* 2012) but also under increasing or replete light conditions in other species (Legrand *et al.,* 1998; Li *et al.,* 1999; Hansen, 2011). Additionally, prey availability has also been shown to be a factor that influences phagotrophy and growth rates in some dinoflagellates (Li *et al.,* 1999; Stoecker, 1999; Berge *et al.,* 2008; Zhang *et al.,* 2013).

Changes in nutrient concentration, light level and prey availability systematically occur during the development of algal blooms (Granéli and Turner, 2006). Furthermore, few studies have addressed the effect of prey type, i.e. different prey species/sizes, on the rates of ingestion in mixotrophs, which is likely to result in different phagotrophic activity within the same species (Legrand *et al.,* 1998). Prey ingestion by harmful algal bloom species may increase their growth rates (Li *et al.,* 1999; Jeong *et al.,* 2005b), facilitate the formation of mono-specific blooms by consuming competitors (Zhang *et al.,* 2013) and reduce prey availability to higher trophic levels (Stoecker *et al.,* 2017; Mitra and Flynn, 2023; Tillmann *et al.,* 2023), potentially altering the persistence and decline of blooms (Flynn *et al.,* 2018). Since the mixotrophic activity of planktonic communities is highly variable and appear to be species specific, it is essential to study the behavior of different harmful blooming taxa to understand their impact on the ecosystem and to better predict their response to the forecasted climate changes (Hallegraeff, 2010).

The aim of our study was to evaluate the effect of nutrient and light availability, as well as prey type, on the phagotrophic activity of mixotrophic bloom-forming dinoflagellates. We compared the prey ingestion of three common harmful dinoflagellate species suggested to have different mixotrophic activity: *Alexandrium minutum*, *H. triquetra* and *P. micans*. The three dinoflagellates were acclimated to different nutrient and light conditions and a set of grazing experiments using three different fluorescently labeled preys was performed. In addition to prey ingestion rates, the effect of the different treatments on cell growth, size, and C and nitrogen (N) cellular content were analyzed. The use of fluorescent acidotropic probes to detect the presence of digestive vacuoles on the three species studied was also evaluated. The results provide new insights into the factors influencing the mixotrophic behavior of bloom-forming dinoflagellates.

## METHOD

### Dinoflagellates strains and culture conditions

*A. minutum* (92 AM2), *H. triquetra* (HT01) and *P. micans* (PM01) were isolated from the Brittany coastal waters (France, North Atlantic), in 2017, 1999 and 1985, respectively. All strains were routinely maintained as non-axenic batch cultures at 17°C, 100 μmol quanta m^−2^ s^−1^ of overhead illumination (Aquatlantis Easy Led FW 6800°K) under a 12:12 h light:dark cycle and grown in sterilized prefiltered natural seawater enriched with K medium (Harrison and Berges, 2005), containing 882 μM nitrate (NO_3_^−^), 50 μM ammonium (NH_4_^+^) and 7 μM Na_2_-glycerophosphate as phosphorous source. In parallel, the dinoflagellate strains were acclimated to low light conditions (15 μmol quanta m^−2^ s^−1^), to low nutrient concentration (K/3 medium: K medium diluted three-fold in seawater; 294 μM NO_3_^−^, 16.6 μM NH_4_^+^ and 2.3 μM Na_2_-glycerophosphate) and to both treatments (low light and K/3) for 3 months before the experiments. The different conditions combined resulted in four different treatments: (1) HL-K: high-light and nutrient replete (regular growing conditions), (2) LL-K: low-light and nutrient replete, (3) HL-K/3: high-light and low-nutrient and (4) LL-K/3: low-light and low-nutrient. After the acclimation period and prior to grazing experiments, growth rates and C and N cellular content were calculated for the three species under the different conditions and tests using fluorescent acidotropic probes were conducted (detailed below).

### Preparation of labeled preys

We used three different prey with different sizes for the experiments: fluorescently labeled bacteria (FLB, ≤ 1 μm), *Synechococcus spp.* (FLS, 1–2 μm) and the small haptophyte *Isochrysis galbana* (FLA, 5–6 μm). Bacteria were isolated from *A. minutum* cultures, they were collected by sequential filtration through 5-, 1.2- and 0.8-μm pore-size polycarbonate membranes (Millipore). *Synechococcus spp.* (RCC2380) and *I. galbana* cultures were routinely maintained as described above (regular growing conditions). The three preys were fluorescently labeled and heat killed as described in Sherr *et al.* (1987) with some modifications explained as followed: cells were concentrated by centrifugation at 5000 g for FLB and FLS and at 800 g for FLA for 12 min, resuspended in 9 mL of phosphate-buffered saline (PBS) adjusted to pH 9 (Gibco, Life Technologies) and 1 mL 4.04 mM DTAF (5-(4,6-dichlorotriazin-2-yl) aminofluorescein, Sigma-Aldrich) and incubated at 60°C in a water bath for 2 h. After incubation, cells were washed at least three times with PBS, resuspended in 0.02 M PPi buffer (solution of tetrasodium pyrophosphate, Sigma-Aldrich, at 0.85% NaCl), briefly sonicated for several 5 s bursts in a bath sonicator (Bioblock Scientific 86 484) at 60% of ultrasound power and stored frozen at −20°C in aliquots. Before each experiment, aliquots were thawed, sonicated for several 5 s bursts and measured through flow cytometry (see below). Labeled preys were used within a month.

### Feeding experiments

For each treatment, a culture of each acclimated dinoflagellate in their exponential growth phase was gently filtered by gravitation through 5-μm nylon membrane (Millipore) to remove associated bacteria from cultures (96–97% of bacteria were removed). Samples for pH, phosphate (PO_4_^3−^) and NO_3_^−^ concentration were taken from the filtrate of the low-nutrient treatments (HL-K/3 and LL-K/3) to verify whether nutrient limitation was present at the time when the experiments were conducted, and analyzed with a segmented-flow autoanalyzer (AA3HR, Seal) using the method of Aminot and Kérouel (2007). Dinoflagellate cells were washed at least three times with 10 mL 0.2-μm-filtered seawater, gently recovered in 180 mL of fresh medium and allowed to settle 24 h before the experiments. Initial dinoflagellate concentration was ~ 1500 cells mL^−1^ for *A. minutum* and *P. micans* and ~ 3000 cells mL^−1^ for *H. triquetra*. All experiments were conducted in the morning at the same hour of the day (3 h after the start of the light phase). For each dinoflagellate and treatment, cultures were divided into 20 mL triplicates and an additional flask with medium was set up as control. Labeled preys were inoculated into each 20 mL triplicate and control flasks. FLS and FLA were added at similar concentrations (1 × 10^6^ and 1 × 10^5^ cells mL^−1^, respectively) that suggested in the literature (Du Yoo *et al.,* 2018; Li *et al.,* 2021). However, studies using FLB in grazing experiments generally added 5–30% of the co-occurring bacterial abundance (Sherr *et al.,* 1987; Sanders and Gast, 2012; Anderson *et al.,* 2018; Costa *et al.,* 2022) or added FLB at the concentrations typical of bacteria in that environment (Christaki *et al.,* 2001; Massana *et al.,* 2006). Based on previous tests (not shown here) that indicated that the three dinoflagellates studied were not feeding upon many bacteria, we opted to eliminate the associated live bacteria (about 10^7^ cells mL^−1^) by several washing steps and added that concentration of FLB (i.e. 1 × 10^7^ cells mL^−1^). Due to an analytical limitation, the initial concentration of FLA in the experiment of *H. triquetra* under the HL-K treatment was 7 × 10^4^ cells mL^−1^. At the beginning of prey addition (t0), dinoflagellate and prey abundance were measured using flow cytometry (detailed below). After 15 min, 1 and 3 h of prey addition, each flask was sampled for cell abundance and ingestion measurements (detailed below). The incubation times were chosen based on previous results on ingestion of labeled preys by mixotrophic protists in order to cover the uptake rates of the different dinoflagellates on the different preys (Chan *et al.,* 2009; Anderson *et al.,* 2017; Costa *et al.,* 2022). Legrand *et al.* (1998) reported ingestion of FLA by *H. triquetra* after 30 min incubation. As far as we know, there was no information about *A. minutum* or *P. micans*. Total bacterial abundance was measured at the beginning and at the end of experiments to ensure negligible initial concentration of associated bacteria (non-labeled and alive) and no growth during the incubations. Associated bacteria accounted for less than 20% of FLB abundance during the experiments. At least 10 mL of culture remained in each flask at the end of the experiment.

### Cell abundances and growth rates

Dinoflagellate, prey and associated bacteria abundances were measured immediately after sampling on a NovoCyte Advanteon flow cytometer (Agilent Technologies) equipped with a blue 488 nm laser, and analyzed with NovoExpress Software (Agilent Technologies). Dinoflagellates were counted directly based on their forward scatter and chlorophyll-based red fluorescence signals. Prey (FLB, FLS and FLA) and total bacteria abundances were counted based on their side scatter and green fluorescence signals. Total bacteria were previously stained with SYBR Green I (Sigma-Aldrich, 1× final concentration) for 10 min in the dark.

Cell-specific growth rates (μ, in d^−1^) were calculated for each time interval using cell abundances as.

$\mathrm{\mu} =\left(\ln\ {A}_n- \ln\ {A}_{n-1}\right)/\left({t}_n- {t}_{n-1}\right).$

where *A_n_* and *A*_*n*-1_ are the cell abundances (in cells mL^−1^) at times *t_n_* and *t*_*n*-1_ (in days), respectively.

### Cellular stoichiometry

Cellular C and N quotas were measured for the three dinoflagellate species under the different treatments after the acclimation period prior to experiments, as well as for the three preys used. After cell abundance measure, culture subsamples (*n* = 3) were filtered through pre-combusted (450°C, 4 h) Whatman 25 mm GF/F glass fiber filters, rinsed three times with 10 mL of 0.2-μm-filtered seawater, placed in glass vials and kept frozen. Prior to analysis, filters were dried at 60°C for 24 h. C and N content on filters were measured by flash combustion/oxidation using an Elemental analyzer (Flash EA, ThermoFisher, Scientific) coupled to a mass spectrometer (Delta plus Thermofisher Scientific) *via* a type III interface. A laboratory weight standard (atropine, certified Elemental Microanalysis) of different masses (8–10 standards) was used to calibrate the analyzer and determine C and N sample content. Analytical precisions obtained from repeated measurements of the certified standard were of ±0.5% and ± 0.04% for C and N content, respectively. Data were normalized to cell counts to obtain mean individual values (in pmol C cell^−1^ and in pmol N cell^−1^).

### Ingestion measurements

Ingested preys were counted through microscopic observation. 2 mL of sample were fixed with Lugol’s solution (0.25% final concentration) followed by 15 μL of 0.1 N sodium thiosulfate to decolorate iodine. Samples were filtered through 3-μm pore-size nylon membranes (Millipore) and dinoflagellate cells were washed and gently recovered in 2 mL of 0.2-μm-filtered seawater. Cells were then stained with 10 μL of calcofluor and gently filtered through 0.8-μm pore-size polycarbonate filters (Whatman). Filters were mounted on glass slides with immersion oil and stored at −20°C until analysis through epifluorescence microscopy within 2–3 months. A total of 100 randomly selected cells per replicate were scanned under BX60 and BX61 Olympus microscopes with 100× objective magnification. Ingested preys and/or digestive vacuoles were visualized using blue light excitation for chlorophyll (algal cells, in red) and fluorescein (prey cells, in green) fluorescence, and UV light excitation for calcofluor fluorescence (theca visualization, in blue). Cell diameters of 20–40 cells were also measured for each dinoflagellate and treatment with an ocular micrometer and used to calculate the equivalent spherical diameters (ESD).

Ingestion rates were calculated from microscopy counts since they provided visual evidence of prey cells inside dinoflagellates, avoiding an overestimation of prey consumption through flow cytometry counts due to attachment of prey items to cell surfaces and to empty theca. Cell-specific ingestion rates (in prey dinoflagellate^−1^ h^−1^) were calculated by dividing uptake rates (prey dinoflagellate^−1^) with the incubation time (in hours) (Sherr *et al.,* 1987). C- and N-specific ingestion rates (in C_prey_ C_dinfolagellate_^−1^ h^−1^ and N_prey_ N_dinfolagellate_^−1^ h^−1^) were calculated using the cell-specific ingestion rates and the C and N cellular content measured for the dinoflagellates and preys in the different conditions. Percentages of feeding cells, i.e. dinoflagellate cells with fluorescent prey and/or vacuoles inside, were calculated as the relative abundance of dinoflagellate cells observed with ingested prey or digestive vacuoles. Clearance rate equivalence (CRe, in nL dinoflagellate^−1^ h^−1^) were calculated dividing cell-specific ingestion rates by average prey concentration (cells nL^−1^) over same incubation time (Rublee and Gallegos, 1989). Daylight C-specific ingestion rates, estimated multiplying hourly rates by 12 (representing daylight hours), were used to estimate the percentage of prey standing stock, in terms of C, consumed by the dinoflagellates population only during daylight hours, since night ingestion rates were not available to estimate daily consumption. For this calculation, the total C of predator and prey populations were estimated assuming the cell abundances used in the experiments of this study.

### Fluorescent acidotropic probes

Two fluorescent dyes commonly used in mixotrophic studies (Carvalho and Granéli, 2006; Anderson *et al.,* 2017; Sato and Hashihama, 2019; Yang *et al.,* 2020; Costa *et al.,* 2022) were tested to quantify the dinoflagellate cells actively feeding. The acidotropic probes are used to stain acidic organelles, including digestive vacuoles, of live cells, and when combined with flow cytometry enable the quantification of cells containing digestive vacuoles, i.e. actively phagotrophic cells. We tested two different acidotropic probes on the acclimated cultures (prior to feeding experiments) of the three dinoflagellates under the different nutrient and light conditions, since cultures were non-axenic and thus with live associated bacteria as potential preys. In order to optimize probes concentration and staining time for our cultured dinoflagellates, LysoTracker Green DND-26 and LysoSensor Yellow/Blue DND-160 (Molecular Probes) were tested at different final concentrations (0.5, 5, 10 and 50 nM for LysoTracker and 0.5, 1 and 2 μM for LysoSensor) and 5 incubation times (2, 5, 10, 15 and 20 min) (Carvalho and Granéli, 2006; Sintes and del Giorgio, 2010). Culture subsamples were incubated with the probes in the dark at room temperature and analyzed through flow cytometry using forward scatter, red and green fluorescence signals. Specificity of the probes was verified through epifluorescence microscopy to ensure staining of digestive vacuoles.

### Statistical analysis

Student’s *t*-test was used to test significant differences between two treatments, and one-way ANOVA with Tukey’s *post hoc* test was applied for comparison among treatments by means of Tukey. Simple linear models were used to capture the relationship between ingestion rates and percentage of feeding cells. All statistics were performed using the R software (4.2.2) and “t.test”, “aov”, “HSD.test” and “lm” functions.

## RESULTS

### Effect of light and nutrient conditions on dinoflagellates growth, cell size and C:N ratio

**Fig. 1:**
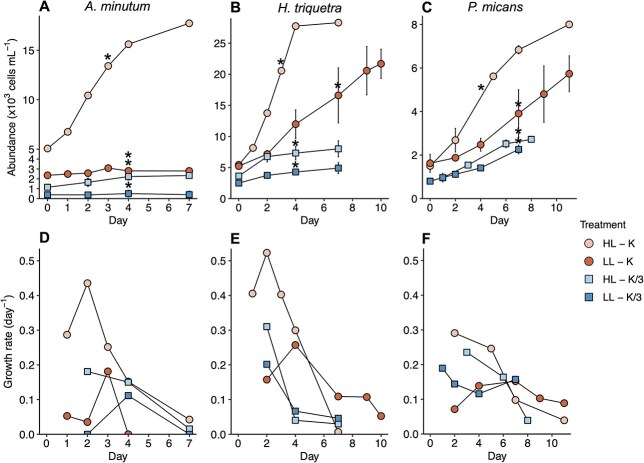
Dinoflagellates growth in the different treatments. (**A**) *A. minutum*, (**B**) *H. triquetra* and (**C**) *P. micans* cell abundances after the acclimation period of cultures. Vertical bars indicate standard deviation from 2 to 3 culture replicates. Asterisks indicate the time when the feeding experiments were conducted. (**D**) *A. minutum*, (**E**) *H. triquetra* and (**F**) *P. micans* specific growth rates (day^−1^) calculated from mean cell abundances. Treatments are indicated in different colors and shapes: HL-K (high-light and nutrient-replete), LL-K (low-light and nutrient-replete), HL-K/3 (high-light and low-nutrients), LL-K/3 (low-light and low-nutrients).

**Fig. 2:**
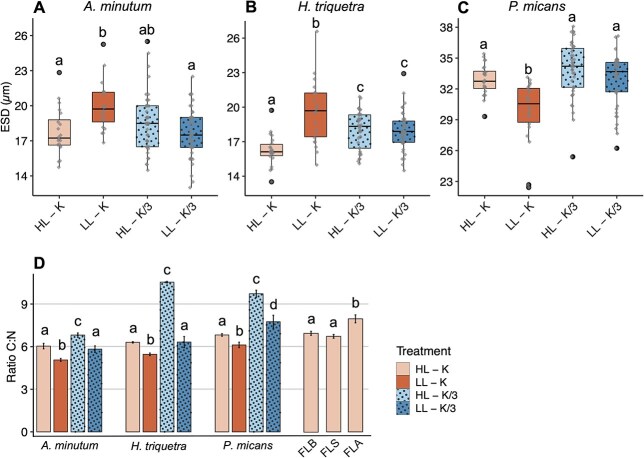
(**A**) *A. minutum*, (**B**) *H. triquetra* and (**C**) *P. micans* cell sizes (ESD, in μm) in the different treatments. Boxplots show median, first and third quartiles and variability outside the quartiles. Black dots indicate outliers and gray diamonds indicate jittered values. Letters indicate significant differences (*P* < 0.05) among treatments (*post hoc* Tukey’s test). (**D**) C:N ratios of the three dinoflagellates in the different treatments and the three preys (FLB: bacteria, FLS: *Synechococcu*s, FLA: *Isochrysis galbana*). Vertical bars indicate standard deviation from three replicates. Treatments are indicated in different colors and patterns. Letters indicate significant differences (*P* < 0.05) among treatments for each dinoflagellate and among preys (*post hoc* Tukey’s test).

Dinoflagellates abundance and growth were monitored after the acclimation period prior to experiments (Fig. 1A-C). All three dinoflagellates grew well under replete conditions (HL-K treatment), reaching the highest specific growth rates on day 2 of culture with 0.43, 0.52 and 0.29 d^−1^ for *A. minutum*, *H. triquetra* and *P. micans*, respectively (Fig. 1D-F). At replete conditions, mean cell size was 17.8 ± 2.0, 16.2 ± 1.3 and 32.9 ± 1.5 μm ESD ± SD (Fig. 2A-C) and cell C:N ratio was 6.1 ± 0.1, 6.3 ± 0.1 and 6.8 ± 0.1 for *A. minutum*, *H. triquetra* and *P. micans*, respectively (Fig. 2D and Supplementary Table S1). All three dinoflagellates decreased its growth in the other treatments respect to replete conditions (Fig. 1). In the low-nutrient treatment alone (HL-K/3) *A. minutum* decreased significantly (*t*-test, *P* < 0.05) its highest growth rate to 0.18 d^−1^. Specific growth rates also decreased significantly (*t*-test, *P* < 0.05) in low light conditions (LL-K and LL-K/3) for the three dinoflagellates, reaching maximum values of 0.18, 0.26 and 0.15 d^−1^ in LL-K, respectively, and to 0.11, 0.20 and 0.19 d^−1^ in LL-K/3, respectively (Fig. 1D-F). Furthermore, under low light conditions alone, cultures achieved their maximum growth rates on days 3–7, later than that observed in the other treatments (Fig. 1D-F).

Light and nutrient conditions changes also affected dinoflagellates cell sizes and C and N cellular content (Fig. 2 and Supplementary Table S1). Low light condition alone (LL-K) significantly influenced the cell sizes of the three dinoflagellates (Tukey’s test, *P* < 0.05). Indeed, *A. minutum* and *H. triquetra* cell sizes increased significantly under this condition whereas *P. micans* cell size decreased (Fig. 2A-C). The three dinoflagellates significantly decreased their cell C:N ratio (Fig. 2D) under low light (Tukey’s test, *P* < 0.001) due to a decrease in C cellular content, and in *A. minutum* and *H. triquetra* also due to an increase in N cellular content (Supplementary Table S1). Under low nutrient conditions, both HL-K/3 and LL-K/3, only *H. triquetra* cell size increased significantly (Tukey’s test, *P* < 0.01) (Fig. 2B). Under the low-nutrient treatment alone (HL-K/3) the three dinoflagellates significantly increased their cell C:N ratio (Tukey’s test, *P* < 0.001) to up to 6.7 (± 0.1), 10.5 (± 0.1) and 9.8 (± 0.1) in *A. minutum*, *H. triquetra* and *P. micans*, respectively (Fig. 2D), related to a higher C cellular content (Supplementary Table S1). Nutrient concentration estimates in low-nutrient treatments revealed that PO_4_^3−^ was rapidly consumed in the first days of culture, decreasing from 1.25 μM (initial medium K/3, day 0) to 0–0.16 μM and 0.26–0.63 μM in HL-K/3 and LL-K/3, respectively, on days 4–7 when the experiments where conducted (Supplementary Table S2). Besides, pH increased in *H. triquetra* and *P. micans* cultures, especially in HL-K/3 treatments where pH was 8.81 and 8.72, respectively, when the experiments were conducted (Supplementary Table S2).

### Use of fluorescent acidotropic probes to detect digestive vacuoles

We obtained positive staining of cells with both LysoTracker and LysoSensor dyes through flow cytometry for the three dinoflagellates; however, we did not see specificity for cellular structures under the epifluorescence microscope. Indeed, we observed alive stained cells under blue and UV light excitation under the microscope using same dye concentrations and incubation times than those used for flow cytometry analysis, but both dyes stained the entire theca structure for the three dinoflagellates, no acidic vacuoles or cellular structures were differentiated (Supplementary Fig. S1). In order to verify the validity of the probes, we used the same protocol for other non-axenic cultures of mixotrophic dinoflagellates such as *Alexandrium tamarense* (with theca), *Karlodinium armiger* and *K. veneficum* (without theca). We distinguished acidic vacuoles under the microscope only in *Karlodinium* species (Supplementary Fig. S1), but not for *A. tamarense* cells, whose entire theca structures were stained. As vacuole staining using acidotropic probes proved ineffective, feeding cells were determined only through the observation of labeled prey within the cells by microscopy (Supplementary Fig. S2).

### Dinoflagellates feeding and cell-specific ingestion rates

**Fig. 3:**
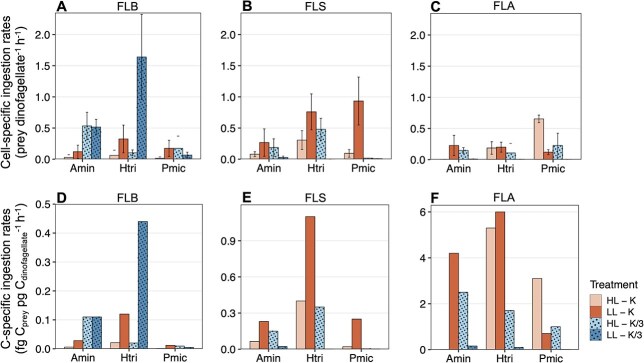
Maximum mean cell-specific ingestion rates of *A. minutum* (Amin), *H. triquetra* (Htri) and *P. micans* (Pmic) on (**A**) FLB (bacteria), (**B**) FLS (*Synechococcus*) and (**C**) FLA (*Isochrysis galbana*) preys. Error bars indicate standard deviation of the three replicates. C-specific ingestion rates of the three dinoflagellates on (**D**) FLB, (**E**) FLS and (**F**) FLA, calculated using mean cell-specific ingestion rates. Note the different scale between plots D and F. Bar colors and patterns indicate the different treatments.

The studied dinoflagellates were feeding on the three preys used in this study, and their phagotrophic activity varied between species and in the different light and nutrient conditions (Fig. 3). The percentage of feeding cells was similar in the three incubation times (15 min, 1 and 3 h) in most cases, except for FLB (Supplementary Fig. S3); however, cell-specific ingestion rates were found maximum at 15 min after prey addition in replete conditions, and at 15 min or 1 h in the other treatments (Supplementary Fig. S4). Indeed, the percentage of feeding cells was positively correlated with cell-specific ingestion rates, but different slopes were observed for the three incubation times (15 min, 1 h and 3 h) (Supplementary Fig. S5).

In replete conditions (HL-K treatment), higher percentages of feeding *A. minutum* and *H. triquetra* cells were found on FLS (*Synechococcus*) (ANOVA, *P* < 0.001), up to 3 (± 1.5) and 6.3 (± 1.3) % of feeding cells, respectively, reaching ingestion rates of 0.08 (± 0.04) and 0.31 (± 0.15) prey dinoflagellate^−1^ h^−1^ (± SD), respectively (Fig. 3B). *P. micans* exhibited significantly higher percentages of feeding cells on FLA (*I. galbana*) (ANOVA, *P* < 0.001), reaching 13 (± 1.0) % of feeding cells and an ingestion rate of 0.65 (± 0.06) prey dinoflagellate^−1^ h^−1^ (Fig. 3C). In low light conditions (LL-K treatment), the number of feeding cells in *A. minutum* and *H. triquetra* cultures increased with decreasing prey size, reaching up to 7.7 (± 0.7) and 18 (± 1.0) % of feeding cells with FLB (bacteria) as prey, respectively, whereas *P. micans* had maximum % of feeding cells of 17.7 (± 2.9) % on FLS as prey (Supplementary Fig. S3). The highest cell-specific ingestion rates were on FLS for the three dinoflagellates, reaching 0.27 (± 0.22), 0.76 (± 0.29) and 0.93 (± 0.38) prey dinoflagellate^−1^ h^−1^ for *A. minutum*, *H. triquetra* and *P. micans*, respectively (Fig. 3B), and were significantly different from other preys in *H. triquetra* (ANOVA, *P* < 0.05) and *P. micans* (ANOVA, *P* < 0.01).

Inorganic nutrients concentrations were measured in the low-nutrient experiments (HL-K/3 and LL-K/3). The results indicate that *P* was the limiting nutrient, PO_4_^3−^ concentration ranging 0–0.63 μM and NO_3_^−^ ranging 278–353 μM (Supplementary Table S2). In the low-nutrient treatment alone (HL-K/3), the preferred type of prey ingested varied between dinoflagellates. The highest cell-specific ingestion rates were 0.53 (± 0.22) prey dinoflagellate^−1^ h^−1^ for *A. minutum* on FLB, 0.48 (± 0.17) prey dinoflagellate^−1^ h^−1^ for *H. triquetra* on FLS and 0.23 (± 0.20) prey dinoflagellate^−1^ h^−1^ for *P. micans* on FLA (Fig. 3A-C), with 13.3 (± 1.7), 9.7 (± 1.3) and 5 (± 2.5) % of feeding cells, respectively (Supplementary Fig. S3). Finally, in the low-light and low-nutrient condition (LL-K/3 treatment), significantly higher percent of feeding cells (ANOVA, *P* < 0.01) and the highest cell-specific ingestion rates (ANOVA, *P* < 0.05) were on FLB for the three dinoflagellates, reaching 15.5 (± 1.5), 31.3 (± 6.3) and 3 (± 1.0) % of feeding cells and 0.52 (± 0.12), 1.64 (± 0.68) and 0.07 (± 0.05) prey dinoflagellate^−1^ h^−1^ for *A. minutum*, *H. triquetra* and *P. micans*, respectively (Fig. 3A-C and Supplementary Fig. S3).

### Phagotrophic C and N prey acquisition, clearance rates equivalence and daylight estimates of C-prey consumption

The preys used, all grown in replete light and nutrient conditions (HL-K), had different C and N cellular content according to cell size (Fig. 2D and Supplementary Table S1). FLS (*Synechococcus*, 1–2 μm) had approximately 4-fold higher C and N than FLB (bacteria, ≤ 1 μm); and FLA (*I. galbana*, 5–6 μm) had 20-fold higher C and N than FLS (Supplementary Table S1). The three dinoflagellates showed higher C- and N-specific ingestion rates when feeding on FLA than on the other smaller preys (Fig. 3D-F and Supplementary Fig. S6) with *A. minutum* and *H. triquetra* reaching 4.2 and 6 fg C_prey_ pg C_dinfolagellate_^−1^ h^−1^, respectively, in the low-light treatment alone, whereas *P. micans* reached 3.1 fg C_prey_ pg C_dinfolagellate_^−1^ h^−1^ in replete conditions (Fig. 3F). When feeding on FLA, *H. triquetra* showed the highest C-based ingestion rates under nutrient-replete conditions (HL-K and LL-K) compared to the two other dinoflagellates, whereas *A. minutum* showed the highest rates in the low-nutrient treatments (HL-K/3 and LL-K/3) (Fig. 3F). When feeding on FLB, *H. triquetra* was the dinoflagellate with the highest C-based ingestion rates in all treatments but in the low-nutrient condition (HL-K/3), where *A. minutum* reached the highest rates compared to the other dinoflagellates (Fig. 3D); and when feeding on FLS, *H. triquetra* also showed the highest C-based ingestion rates in all conditions but in the co-limitation treatment (LL-K/3), where *A. minutum* reached the highest values (Fig. 3E).

**Fig. 4:**
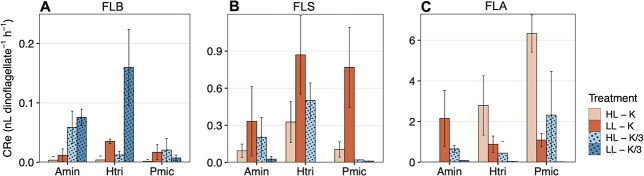
Maximum mean clearance rates equivalence (CRe) of *A. minutum* (Amin), *H. triquetra* (Htri) and *P. micans* (Pmic) on (**A**) FLB (bacteria), (**B**) FLS (*Synechococcus*) and (**C**) FLA (*Isochrysis galbana*) preys. Error bars indicate standard deviation of the three replicates. Colors and patterns indicate the different treatments.

Clearance rate equivalence estimates (CRe), which indicate the prey size-dependent feeding and competition for prey, expressed as volume cleared of prey, increased with increasing prey size (Fig. 4). The results show that the three studied dinoflagellates had the highest CRe on FLA: *H. triquetra* and *P. micans* cleared, respectively, up to 2.8 and 6.3 nL dinoflagellate^−1^ h^−1^ in replete conditions, and *A. minutum* up to 2.2 nL dinoflagellate^−1^ h^−1^ in low-light conditions (LL-K treatment) (Fig. 4C). When feeding on FLS, the three dinoflagellates had the highest CRe under low-light conditions (LL-K treatment), reaching 0.3, 0.9 and 0.8 nL dinoflagellate^−1^ h^−1^ for *A. minutum, H. triquetra* and *P. micans*, respectively (Fig. 4B) (ANOVA *P* > 0.05, *P* < 0.01, *P* < 0.01 for *A. minutum, H. triquetra* and *P. micans*, respectively). The highest CRe on FLB were below 0.2 nL dinoflagellate^−1^ h^−1^ for the whole set of experiments (Fig. 4A).

Using C-specific ingestion rates and considering only the daylight phagotrophic rates (the activity during the 12 h of light, period when the experiments were conducted), we estimated that *H. triquetra* and *P. micans* populations can consume, respectively, up to 14 and 13% of C-*I. galbana* (FLA) standing stock in replete conditions during daylight period, lowering their consumption to less than 5% in low-nutrient and low-light conditions. However, *A. minutum* population can consume during the daylight period up to 6.5% of C-*I. galbana* in low-light nutrient-replete conditions and less than 1% in the other treatments during the daylight hours. When considering *Synechococcus* (FLS) as prey, the three dinoflagellates showed higher percentage of daylight consumption in low-light nutrient-replete conditions, under which we predict that *A. minutum*, *H. triquetra* and *P. micans* can consume during the daylight period up to 0.9, 3.5 and 1.5% of C-*Synechococcus* standing stock, respectively. Using bacteria as prey (FLB), we predict that *A. minutum* and *H. triquetra* populations consume during the daylight period from <0.03% of the bacterial C standing stock in replete conditions to 0.14 and 0.50%, respectively, in low-light and low-nutrient conditions (Supplementary Fig. S7).

## DISCUSSION

The phagotrophic activity of harmful bloom-forming dinoflagellates is likely to have important ecological implications on the C and nutrient acquisition, and therefore survival, of these species during non-bloom periods and during the initiation, development and decline of bloom events (Burkholder *et al.,* 2008; Flynn *et al.,* 2018), since these events entail important changes in nutrient and light conditions, composition and concentration of potential preys and in grazing/viral pressures (Glibert *et al.,* 2018). Our results show that the role of phagotrophy as an alternative mode of nutrition varies under different nutrient concentrations, light availability and type/size of prey, and that the influence of these parameters depends on the dinoflagellate species. As far as we know, this study provides first ingestion rates measures, quantified through observation of ingested prey, for several bloom-forming dinoflagellates under the influence of different nutrient and light conditions and their phagotrophic response to different types of prey.

### Influence of nutrient and light availability on dinoflagellates fitness and phagotrophic activity

Light availability and nutrient concentration are main factors controlling phytoplankton growth and, in turn, community composition (Sunda and Huntsman, 1997; Marañón *et al.,* 2015; Latasa *et al.,* 2016; Lee *et al.,* 2017). Our results show that a lower light availability (from 100 to 15 μmol quanta m^−2^ s^−1^) decreased by half the highest specific growth rates of the three dinoflagellates. Moreover, the maximum growth was reached later compared to replete conditions in the three dinoflagellates. These results suggest that lower light availability limited C fixation through photosynthesis, supported by the decrease in the C:N cellular ratio, which resulted in a slower growth compared to the other treatments. Lower light availability translated to a significant increase in cell size in *A. minutum* and *H. triquetra* (Fig. 2), in agreement with the observed inverse relationship between growth and cell size with light limitation (Sunda and Huntsman, 1997). Contrarily, *P. micans* decreased its cell size in low-light conditions with respect to the other treatments, in disagreement with the measured growth rates, since studies have reported that when growth rates are similar, low light leads to the dominance of smaller cells, more efficient in light absorption (Finkel *et al.,* 2010; Marañón *et al.,* 2012).

Growth rates decreased under low-nutrient conditions compared to replete conditions in the three dinoflagellates, although reaching the maximum growth on the same day of culture. Both *A. minutum* and *H. triquetra* showed higher cell-specific ingestion rates under co-reduced nutrient and light conditions compared to the other treatments, with *H. triquetra* having ingestion rates 3-fold higher than *A. minutum*. Nutrient and/or light limitation have shown to also increase ingestion rates of other photo-phagotrophic protists (Legrand *et al.,* 1998; Smalley *et al.,* 2003; Li *et al.,* 2021; Koppelle *et al.,* 2022). All cultures had always bacteria as potential living prey (non-axenic cultures); however, in resource-reduced conditions (lower light, lower nutrients or both), *A. minutum* had higher difficulties acclimating and maintaining (almost without increasing in abundance) than *H. triquetra*, with limited flexibility to light or nutrient changes. Other studies have also revealed that the addition of prey under different nutrient conditions had no effect on the growth of other *Alexandrium* species (Zhang *et al.,* 2013). This, together with the lower ingestion rates on the studied preys, suggests that *A. minutum*, although capable of phagocytizing different preys, might be mainly autotroph, relying on photosynthesis as the main support for growth, and that phagotrophy would not be enough to maintain growth or increase abundance for many days in deficient conditions. However, it should be noted that varying mixotrophic capabilities have been observed among different *Alexandrium* strains (Blossom *et al.,* 2012) and, moreover, a loss of mixotrophy has been found in *Alexandrium* strains after three years under autotrophic culture conditions (Blossom and Hansen, 2021). Considering that the strain utilized in this study was isolated 4 years before and maintained under replete conditions, it is plausible that its phagotrophic capability may have been diminished. It is also to note that *A. minutum* is known to be allelopathic (Arzul *et al.,* 1999) and to induce the lysis of competitors (Long *et al.,* 2021). Therefore, the combination of allelopathic lysis with the osmotrophy of dissolved organic matter might be an alternate trophic strategy. For *H. triquetra*, phagotrophy may be a nutritional strategy to maintain its growth when conditions are deficient for photosynthesis and/or osmotrophy, also observed by Legrand *et al.* (1998).

*P. micans* growth was less affected by a lower nutrient availability compared to lower light, and highest specific growth rates only decreased ca. 20% in low-nutrient treatments with respect to replete conditions. Other studies have reported higher prey ingestion of *Prorocentrum* species under nutrient limitation (Stoecker *et al.,* 1997; Johnson, 2015); however, although similar ingestion rates than *P. minimum* were obtained when fed on a cryptophyte under P limitation (Johnson, 2015), our results do not indicate increased phagotrophy under lower or deficient nutrient availability in *P. micans*. In low-nutrient treatments, dinoflagellates were likely under P stress (N:P ratio > 500 and PO_4_^3−^ concentration of 0–0.6 μM) (Mayers *et al.,* 2014), although P limitation cannot be ensured since the provided P source was organic (Na_2_-glycerophosphate) and the nutrient analyses indicate only the available PO_4_^3−^ at that moment. Although the P cellular content was not measured, the nutrient concentration decrease in the medium resulted in a slight increase in cell size compared to replete conditions (Fig. 2), supporting a P limitation in low-nutrient treatments (John and Flynn, 2002). Furthermore, in low-nutrient treatments, especially under high light (HL-K/3), *H. triquetra* and *P. micans* could also have experienced a growth rate decrease due to pH stress, considering that it has been reported that the growth rate of *H. triquetra* and *P. minimum* declined by ~ 20% at pH 8.8–8.9 (Hansen, 2002), which were the values reached in our cultures (pH 8.8 and 8.7 in *H. triquetra* and *P. micans*, respectively, under HL-K/3). The pH stress could explain the lack of phagotrophy responses by *P. micans* under lower nutrient conditions.

On the other hand, *P. micans* was the species with the highest cell-specific ingestion rates (up to 0.65 prey dinoflagellate^−1^ h^−1^) under replete nutrient and light conditions, suggesting that *P. micans* complement phototrophy with phagotrophy to grow even in non-deficient conditions. This is a similar rate reported for *P. pervagatum* feeding on a cryptophyte (Tillmann *et al.,* 2023). Nonetheless, *P. micans* also appears to be primarily photosynthetically dependent, with ingestion rates well below other known highly phagotrophic dinoflagellates such as *K. mikimotoi* (ingesting up to 86 bacteria dinoflagellate^−1^ h^−1^ under replete nutrient and light conditions) (Zhang *et al.,* 2013).

### Effect of prey type on the phagotrophic activity and estimates of diurnal prey consumption

Our results show that under lower light availability the three dinoflagellates had higher cell-specific ingestion rates on *Synechococcus* (FLS), and that under low-nutrient conditions (for *A. minutum*) and/or co-reduced light and nutrient conditions (for the three dinoflagellates) they had higher cell-specific ingestion rates on bacteria (FLB) compared to the other preys. However, the three dinoflagellates showed higher C- and N-specific ingestion rates and CRe on *I. galbana* (FLA) in all treatments (Figs 3 and 4), likely related to the different size, stoichiometry, C and N cell content and concentration of the three preys tested (Baer *et al.,* 2017).

The prey preferences observed in response to the different limiting resource conditions may be linked to variations in prey quality, which has been demonstrated to influence the elemental composition of phagotrophic flagellates (Zhang *et al.,* 2017). Our results suggest that the studied dinoflagellates can supplement nutrient requirements by phagocytizing bacteria when nutrients are scarce; however, when photosynthesis, and thus C fixation, is limited by low light availability alone, *Synechococcus* is the preferred prey among the three tested to supplement C demand. However, even though cell-specific ingestion rates were higher for a specific prey according to the limiting conditions, the higher acquisition of C and N per dinoflagellate cell was consistently with the largest prey (*I. galbana*, FLA). Whereas cell-specific ingestion rates indicate the relative success of the studied dinoflagellates as phagotrophs, regardless of prey concentration, CRe indicate competition for prey taking into account the effect of prey density (Edwards *et al.,* 2023), and is influenced by the rate of predator–prey encounters and other aspects related to nutrition mechanisms that favor prey capture (Nielsen and Kiørboe, 2021). Despite having fewer encounters with *I. galbana,* given its concentration was 1 to 2 orders of magnitude lower than the smaller preys (bacteria and *Synechococcus*), the dinoflagellates acquired higher C and N feeding on a lower number of ingested prey. Besides, prey saturation is likely in our experiments with FLA. The higher CRe values in *H. triquetra* with FLA under HL-K treatment, which was conducted using a lower prey concentration (see Method section), indicate prey saturation, given that lower prey concentration did not translate into a change in the cell-specific ingestion rates. Overall, our findings support that, while phagotrophic dinoflagellates are capable to selectively choose their prey based on nutrient and C requirements (Hansen and Calado, 1999; Liu *et al.,* 2022), they consume prey biomass more efficiently on the larger prey.

Predator–prey imbalances, caused by temperature, light and nutrient concentration changes, as well as virus-host interactions, are thought to be the factors controlling the initiation, maintenance and decline of blooms (Irigoien *et al.,* 2005; Behrenfeld and Boss, 2014; Flynn *et al.,* 2022). During the development of algal blooms, communities can be exposed to light and/or inorganic nutrients limitation due to the increase of algal biomass causing the bloom. Current evidence regarding phagotrophic dinoflagellates generally indicates an ingestion decrease during the night period or in dark conditions for many species (Li *et al.,* 1999; Berge *et al.,* 2008; Kim *et al.,* 2008; Ng *et al.,* 2017; Arias *et al.,* 2020); however, studies on the natural diel cycles of phagotrophic activity in mixotrophic dinoflagellates is still limited, and diel feeding patterns may vary among species and in response to environmental changes (Stoecker *et al.,* 1997; Smalley *et al.,* 2012). Here, we cannot estimate daily consumption rates by the dinoflagellates studied since our experiments do not cover the day-night cycle. However, assuming phagotrophic activity only during daylight hours (12 h), *H. triquetra* and *P. micans* would still cause a loss of ~ 13% of small flagellates (~6 μm) standing stock biomass during the daylight period in replete light and nutrient conditions, which likely have a significant impact on the community, both under bloom and non-bloom conditions, reducing prey availability to heterotrophic predators. In low light conditions, the dinoflagellates studied here can be responsible of up to 3.5–6.5% of small flagellates and 1–3.5% of *Synechococcus* standing stock biomass consumption during the daylight period, similar to the estimated for the small algae *Florenciella* (Li *et al.,* 2021). However, it is important to consider that these estimates may vary according to changes in the abundance of predators and prey. Although the ingestion rates measured on bacteria are not likely to have a relevant impact on the bacterial community (consuming less than 1% of bacteria standing stock per day), the acquisition of N and P from phagotrophy on bacteria are found to be significant despite the low ingestion rates (Mitra and Flynn, 2023) and could play a role during blooms, helping to maintain them when light or nutrients are limiting (Burkholder *et al.,* 2008; Koppelle *et al.,* 2022).

### Methodological considerations when estimating ingestion rates

As far as we know, only four studies have reported prey ingestion rates by the dinoflagellates species studied here. Some discrepancies exist in the reported ingestion rates, and there are various methodological factors that should be considered when comparing these results. Legrand *et al.* (1998) examined *H. triquetra* phagotrophy on fluorescently labeled *Synechococcus*, a small flagellate (3 μm) and the diatom *Thalassiosira pseudonana* (6 μm) under replete and deplete N and P and under light (100 μmol quanta m^−2^ s^−1^) and dark conditions. Contrary to this study, they did not report ingestion on *Synechococcus* in any condition. However, they observed ingestion of the other larger preys under nutrient deficiency, both under light and dark conditions at the same rate. The maximum ingestion rate of *H. triquetra* on FLA (~6 μm) measured in our study (0.2 prey dinoflagellate^−1^ h^−1^) is within the range of values reported by these authors (0.06–0.4 prey dinoflagellate^−1^ h^−1^).

Jeong *et al.* (2005b) measured grazing of *H. triquetra* and *P. micans* on a live unidentified cryptophyte, similar in size to *I. galbana*, under replete nutrients and low light (20 μmol quanta m^−2^ s^−1^) conditions. They obtained ~ 0.09 and 0.1 prey dinoflagellate^−1^ h^−1^ for *H. triquetra* and *P. micans*, respectively, values comparable to those obtained by Legrand *et al.* (1998) and by this study. Jeong *et al.* (2005a) reported ingestion on live *Synechococcus* by the same dinoflagellates as in our study in nutrient-replete and low light (30 μmol quanta m^−2^ s^−1^) conditions, but they reported higher cell-specific ingestion rates (3.2, 4.4 and 35.4 prey dinoflagellate^−1^ h^−1^ for *A. minutum*, *H. triquetra* and *P. micans*, respectively) under similar nutrient, light and prey concentration conditions. Finally, Zhang *et al.* (2013) obtained ingestion rates of *P. micans*, under 45 μmol quanta m^−2^ s^−1^ and from replete to deplete nutrient conditions, ranging from ~ 0.1 to 0.9 prey dinoflagellate^−1^ h^−1^ on *I. galbana* as live prey, which are comparable to this study, and from ~ 1 to 10 prey dinoflagellate^−1^ h^−1^ on bacteria as live prey, values much higher than the obtained in this study under similar conditions (<0.2 prey dinoflagellate^−1^ h^−1^).

The studies mentioned above (Jeong *et al.,* 2005a, 2005b; Zhang *et al.,* 2013) are based on prey disappearance to measure prey ingestion, relying on changes in prey concentration during incubations of 3 to >10 days. However, there are other processes, in addition to phagotrophy, that can induce the disappearance of prey in cultures that should be considered, such as allelopathy, discussed by Zhang *et al.* (2013). Allelopathy is the release of secondary metabolites, i.e. allelochemicals, that can produce negative effects on co-occurring protists, such as growth and photosynthesis inhibition (Granéli and Hansen, 2006). The production of allelochemicals can vary according to environmental stress and between strains of same species, as has been observed for different *A. minutum* strains (Long *et al.,* 2018). We tested the allelopathy of the *A. minutum* strain used in this study on live *Synechococcus* by measuring the maximum photosystem II quantum yield (F_v_/F_m_, as an estimate of photosynthetic activity) of *Synechococcus* when exposed to *A. minutum* filtrate following Long *et al.* (2018). The results revealed that *A. minutum* exudates decreased and inhibited the photosynthetic capacity of *Synechococcus* and their cell concentration by ~ 1.5–2 fold (data not shown). Thus, at least for *A. minutum*, quantifying phagotrophy through prey disappearance with live *Synechococcus* could have been overestimated due to allelopathic interactions. It is crucial to acknowledge this factor when co-culturing with algae species (Fistarol *et al.,* 2004; Prince *et al.,* 2008). In addition, microscopic observations revealed that many prey items were attached to theca rather than being inside cells, and under low nutrient conditions the studied dinoflagellates produced mucus, a feeding strategy reported in other *Prorocentrum* and *Alexandrium* species (Blossom *et al.,* 2012; Tillmann *et al.,* 2023) that results in numerous prey items attached to the mucus trap. Considering these observations, there is a risk of overestimating phagotrophy if preys attached to theca or mucus are erroneously considered ingested based solely on prey disappearance estimates.

Nevertheless, results based on microscopy counts and dead labeled preys have also some limitations. The results in this study revealed that the highest cell-specific ingestion rates were in general at 15 minutes after prey addition, despite that the percentage of feeding dinoflagellates was similar in the three incubation times measured (15 min, 1 and 3 h). This could be explained by the fact that after 1–3 h some prey items could have been already digested and their fluorescence blurred, complicating its detection. Previous studies on phagotrophic protists have estimated digestion times of ~1 h for bacteria (Sherr *et al.,* 1988) and for *Synechococcus* (Dolan and Šimek, 1988). Moreover, DTAF-stained bacteria can exhibit serious bleaching from 5 minutes under blue light (Sherr *et al.,* 1987), potentially affecting FLB visualization under epifluorescence microscopy given that the analysis lasted between 15 min and 1 h for each sample. In addition, it is difficult to estimate the real number of bacteria inside digestive vacuoles, and large predator cells with high red fluorescence intensity, such as *P. micans* (~32 μm), may also hinder the detection of small preys such as bacteria (~1 μm), underestimating their real ingestion. Taking this into account, the lower impact of nutrient and/or light decrease on *P. micans* growth rates could also be explained by the ingestion of associated living bacteria present in the cultures, underestimated in our results. It should also be considered the possible underestimation of our values due to the use of surrogate prey (dead) in our experiments, as there could be a rejection from predators or a preference for living prey (Bock *et al.,* 2021). In any case, the measurements in this study reflect actual prey uptake, and longer incubation times (>3 h or days, using live prey) and night ingestion estimates are needed to have a more comprehensive understanding of prey consumption over time and its impact on growth rates.

In this study we also tested two fluorescent acidotropic probes. These dyes label acidic organelles in live cells and are commonly used to identify mixotrophs in natural communities (Anderson *et al.,* 2017; Sato and Hashihama, 2019; Choi *et al.,* 2020) or to quantify feeding protists in experimental studies (Carvalho and Granéli, 2006; Bowers *et al.,* 2010; Anderson *et al.,* 2018; Yang *et al.,* 2020). However, the results obtained here on several phagotrophic dinoflagellates question their reliability when applied to natural communities and highlight the importance of visually verify their specificity to avoid misleading results (Wilken *et al.,* 2019).

## CONCLUSIONS

Phagotrophy is a nutritional mode that supports carbon and nutrient acquisition of a primarily photosynthetic lifestyle in *A. minutum*, *H. triquetra* and *P. micans*. The three dinoflagellates responded differently to light and nutrient availability changes in terms of growth, cell size, phagotrophic activity and prey preference, showing different trophic flexibility and competitiveness depending on the environmental conditions. The phagotrophic activity of dinoflagellates appears to be influenced by both size and quality of prey, suggesting their ability to selectively choose prey types based on growth limitations. The results of this research suggest that phagotrophy could be of advantage in short periods of light or nutrient limitation and may play different roles during the development of blooms of the three dinoflagellates studied. The mixotrophic feeding of *H. triquetra* and *P. micans*, showing higher prey ingestion compared to *A. minutum*, could have an impact on small flagellates and *Synechococcus* populations, and therefore implications for the energy transfer through the food web. While cell- or C-specific ingestion rates serve as useful indicators of phagotrophic dynamics, accurately estimating the ingestion rates in terms of C or N fluxes, i.e. the C or N actually used from preys, requires the use of stable isotope labelling. Future research focused on the quantification of C, N and P uptake through photosynthesis, phagotrophy and osmotrophy is required to further understand the nutritional strategy and the effect of changing environmental conditions. Stable isotope labelling, double FISH and nanoSIMS are promising techniques that combined will help to quantify bulk and single-cell mixotrophic activities of cultured and natural plankton communities (Beisner *et al.,* 2019).

## Supplementary Material

Supplementary_material_phagotrophy_revised_fbae038
