# Supplementary material for: Impact of light and nutrient availability on the phagotrophic activity of harmful bloom-forming dinoflagellates
Source: J Plankton Res. 2024 Jul 17;47(1):fbae038. doi: 10.1093/plankt/fbae038 (PMC11774208; doi:10.1093/plankt/fbae038)
Supplement: Supplementary_material_phagotrophy_revised_fbae038 [file supplementary_material_phagotrophy_revised_fbae038.pdf]

Impact of light and nutrient availability on the phagotrophic activity of harmful bloom-forming dinoflagellates

Catalina Mena*, Marc Long, Ophélie Lorand, Pascale Malestroit, Emilie Rabiller, Jean-François Maguer, Stéphane L'Helguen and Aurore Regaudie de Gioux

*Corresponding author: catalina.mena@ieo.csic.es. Present address: Centre Oceanogràfic de Balears (COB-IEO, CSIC), Moll de Ponent s/n, 07015 Palma (Illes Balears), Spain.

SUPPLEMENTARY MATERIAL

Figure S1

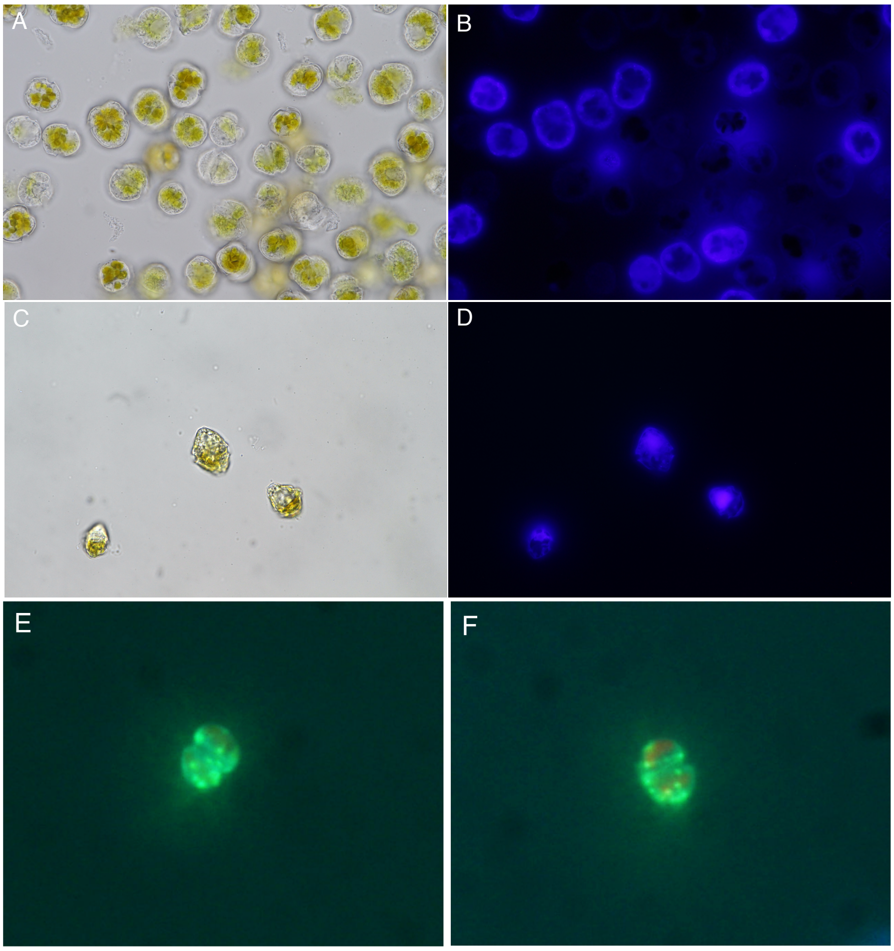

Fig. S1. (**A, B**) *A. minutum* and (**C, D**) *H. triquetra* cells stained with LysoSensor Yellow/Blue DND-160 (Molecular Probes) at 1 µM final concentration and 5 min incubation under (A, C) bright field and (B, D) UV light excitation. No acidic vacuoles were detected, the entire cell was stained. (**E, F**) *Karlodinium veneficum* cells stained with LysoTracker Green DND-26 (Molecular Probes) at 10 nM final concentration and 5 min incubation under blue light excitation.

Figure S2

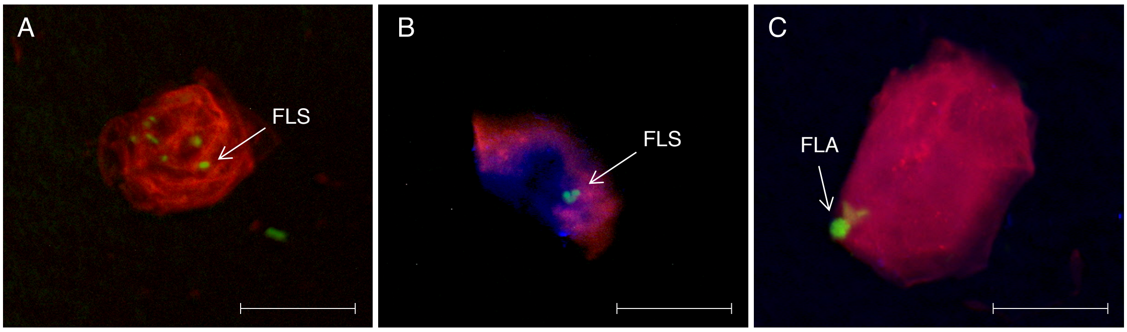

Fig. S2. (**A**) *A. minutum*, (**B**) *H. triquetra* and (**C**) *P. micans* cells with ingested labelled preys. Images were taken using a Vivatome microscope under blue and UV excitation. Preys, indicated with arrows, are seen as green-fluorescent inclusions, whereas red-fluorescence indicate chlorophyll. The use of calcofluor allowed to delimit the cell theca and thus to identify if preys were outside or inside algal cells (not shown here). Scale bars indicate 20 µm.

Figure S3

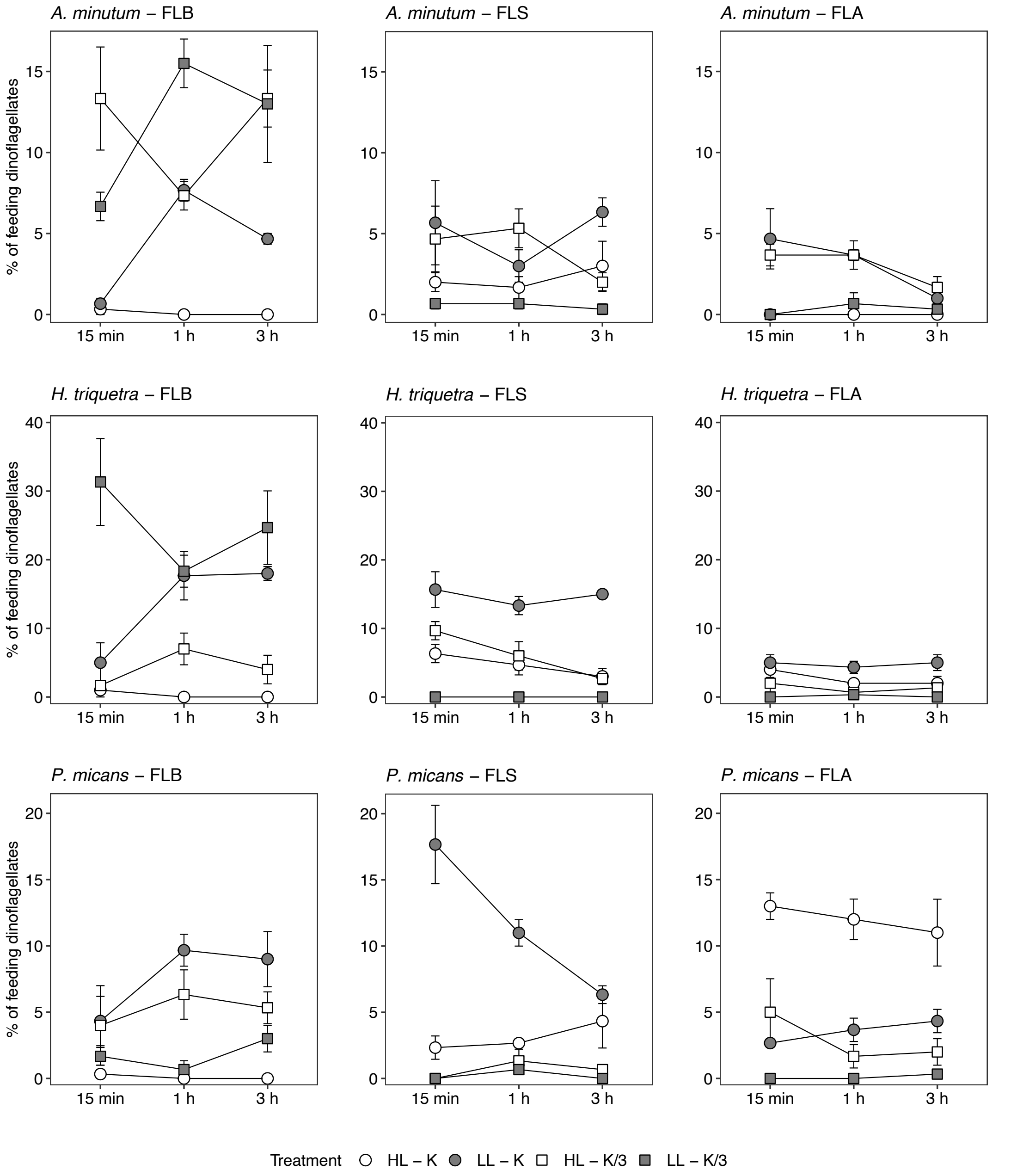

Fig. S3. Percentage of feeding dinoflagellates on FLB, FLS and FLA at the three sampling times (15 min, 1h and 3h) in the different treatments, indicated in different shapes and colors. Mean values from the three replicates are shown, and error bars indicate the standard error.

Figure S4

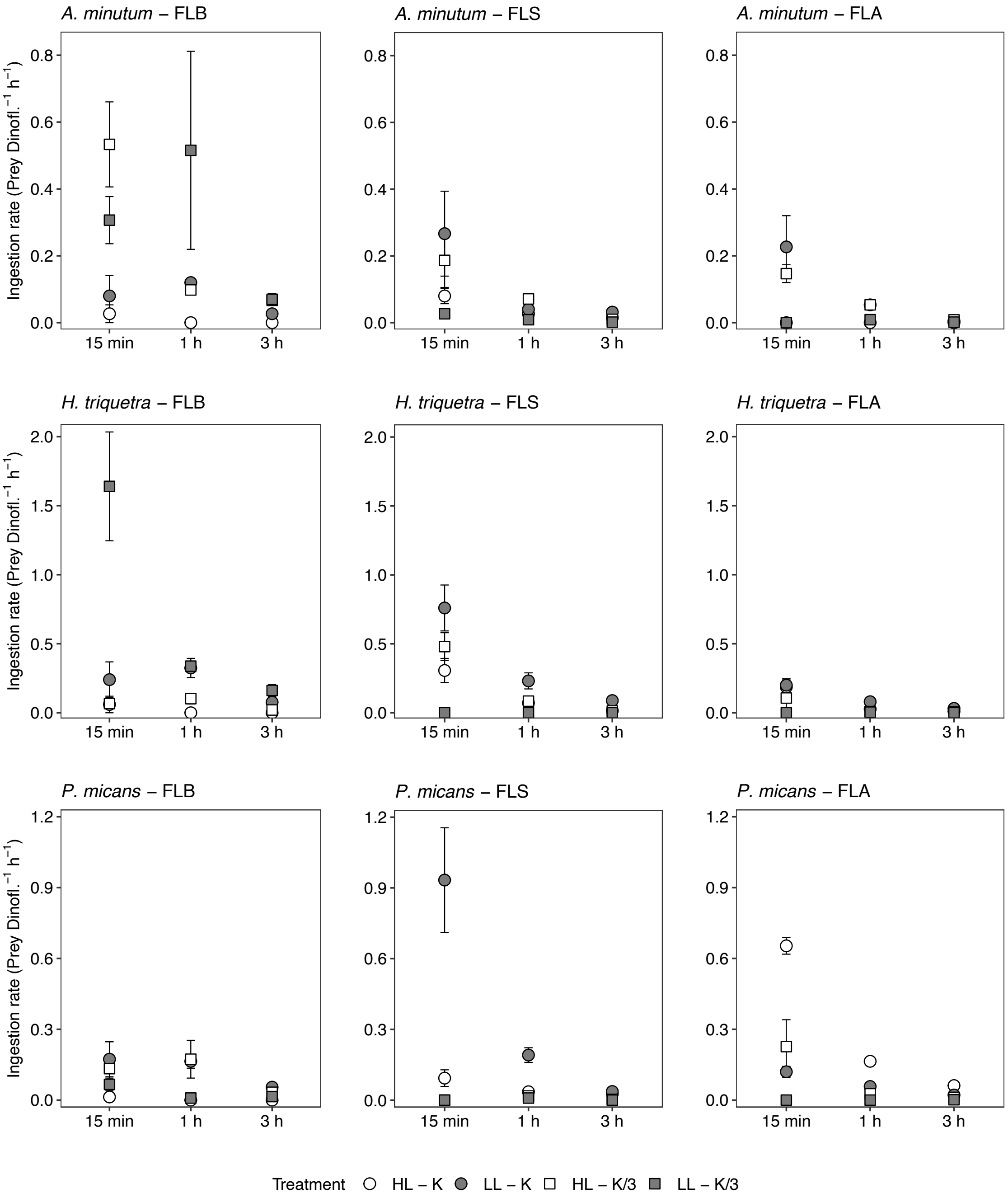

Fig. S4. Cell-specific ingestion rates on FLB, FLS and FLA at the three sampling times (15 min, 1h and 3h) in the different treatments, indicated in different shapes and colors. Mean values from the three replicates are shown, and error bars indicate the standard error.

Figure S5

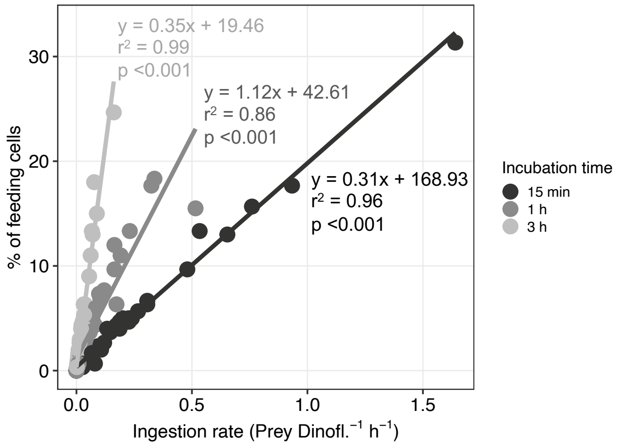

Fig. S5. Relationship between cell-specific ingestion rates and percentage of feeding cells at each incubation time: 15 min, 1h and 3h. The slope, r^2 and p value are indicated for each linear model.

Figure S6

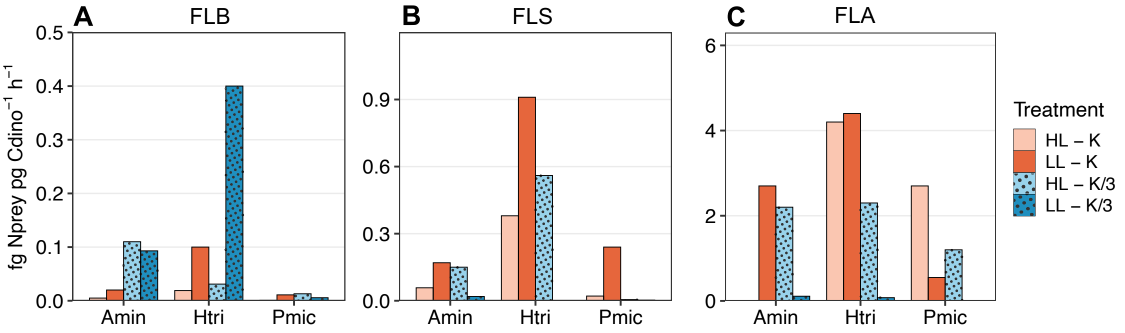

Fig. S6. N-specific ingestion rates of the three dinoflagellates on (**A**) FLB, (**B**) FLS and (**C**) FLA, calculated using maximum mean cell-specific ingestion rates (main Fig. 3). Note the different scale between plots. Bar colours and patterns indicate the different treatments.

Figure S7

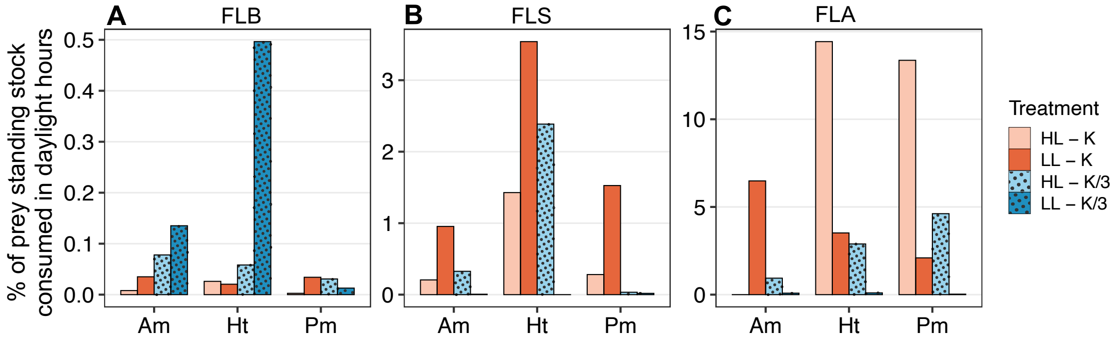

Fig. S7. Estimates of the percentage of prey standing stock consumed during the daylight period by *A. minutum* (Am), *H. triquetra* (Ht) and *P. micans* (Pm) populations on (**A**) bacteria (FLB), (**B**) *Synechococcus* (FLS) and (**C**) *I. galbana* (FLA) in the different treatments, using CRe data and considering 12h of diurnal activity. Treatments are indicated in different colours and patterns.

Table S1. Mean cellular C and N content (n = 3) and mean cell sizes (ESD) (n = 20-40) of the three dinoflagellates in the different treatments and of the three preys used.

Species	Treatment	C content (pmol C $cell^{-1}$ ± SD)	N content (pmol N $cell^{-1}$ ± SD)	ESD (µm ± SD)
A. minutum	HL – K	59.51 (± 0.76)	10.01 (± 0.24)	17.8 (± 2.0)
	LL – K	56.65 (± 2.70)	11.18 (± 0.43)	19.9 (± 2.1)
	HL – K/3	61.43 (± 2.07)	9.02 (± 0.22)	18.6 (± 2.6)
	LL – K/3	59.56 (± 5.05)	10.37 (± 0.89)	17.7 (± 2.1)
H. triquetra	HL – K	37.14 (± 1.81)	5.89 (± 0.26)	16.2 (± 1.3)
	LL – K	35.13 (± 1.32)	6.05 (± 0.33)	19.6 (± 2.7)
	HL – K/3	66.59 (± 1.11)	6.18 (± 0.14)	18.0 (± 1.6)
	LL – K/3	48.73 (± 7.32)	7.68 (± 0.78)	17.9 (± 1.7)
P. micans	HL – K	221.16 (± 12.81)	32.41 (± 1.85)	32.9 (± 1.5)
	LL – K	181.62 (± 2.11)	28.70 (± 0.62)	29.9 (± 3.0)
	HL – K/3	237.60 (± 20.12)	24.43 (± 1.91)	33.9 (± 2.7)
	LL – K/3	170.33 (± 12.39)	21.95 (± 0.41)	32.9 (± 2.6)
Bacteria (FLB)	HL – K	0.013 (± 0.001)	0.002 (± 0.000)	0.7-1*
Synechococcus (FLS)	HL – K	0.048 (± 0.004)	0.007 (± 0.001)	1-2*
I. galbana (FLA)	HL – K	1.055 (± 0.035)	0.132 (± 0.008)	5-6*

* Prey sizes obtained from literature: Zhang et al. 2013; Rublee and Gallegos 1989; Yoo et al. 2018; Jeong et al. 2005.

Table S2. Inorganic nutrient concentration (n = 2) and pH measured for the K and K/3 mediums used for cultures and in the low nutrient treatments during the experiments, which were taken at exponential phase when grazing experiments where performed.

Species	Treatment	NO_2^- (µM ± SD)	NO_3^- (µM ± SD)	PO_4^{3-} (µM ± SD)	pH
K	-	-	919.85 (± 53.53)	4.36 (± 0.08)	-
K/3	-	0.13 (± 0.08)	299.32 (± 18.34)	1.25 (± 0.00)	8.04
A. minutum	HL – K/3	0.20 (± 0.02)	334.13 (± 4.44)	0	8.08
	LL – K/3	0.23 (± 0.01)	350.55 (± 17.04)	0.26 (± 0.09)	8.02
H. triquetra	HL – K/3	0.51 (± 0.02)	306.61 (± 10.87)	0.12 (± 0.06)	8.81
	LL – K/3	0.05 (± 0.02)	353.46 (± 9.79)	0.34 (± 0.05)	8.42
P. micans	HL – K/3	0.11 (± 0.01)	278.01 (± 2.65)	0.16 (± 0.14)	8.72
	LL – K/3	0.23 (± 0.01)	330.35 (± 2.95)	0.63 (± 0.06)	8.51
